# Effects of fruit and vegetable waste addition on corn stalk silage quality

**DOI:** 10.5713/ab.24.0106

**Published:** 2024-06-25

**Authors:** Li Li Wang, Yan Fen Li, Li Zhuang Wu, Young Sang Yu, Xaysana Panyavong, Jong Geun Kim

**Affiliations:** 1 Research Institute of Eco-friendly Livestock Science, Institute of GreenBio Science Technology, Seoul National University, Pyeongchang 25354, Korea; 2 Graduate School of International Agricultural Technology, Seoul National University, Pyeongchang 25354, Korea

**Keywords:** Corn Stalk, Fruit and Vegetable Waste, Silage Quality

## Abstract

**Objective:**

In this study, we explored the effect of fruit and vegetable waste addition on the quality of corn stalk silage.

**Methods:**

Corn stalks were ensiled 20 days after ear harvesting and mixed with fruit and vegetable waste (FVW) consisting of apple, orange, broccoli, and Chinese cabbage waste as 3% of fresh matter. Fruit waste consisted of solid residue obtained after juicing, and vegetable waste was collected from farms and cut into small pieces (2 to 3 cm). The materials were stored anaerobically in 20-L silo buckets and opened after 60 days of fermentation.

**Results:**

There were significant differences in dry matter (DM), acid detergent fiber (ADF), neutral detergent fiber (NDF), total digestible nutrient (TDN), and relative feed value (RFV) levels in FVW derived from all tested raw materials (p<0.05). Corn stalk mixed with orange waste (CSOW) had the highest DM content (28.77%), lowest ADF and NDF content (47.78% and 26.62% of DM, respectively), and highest TDN and RFV content (69.21 and 133, respectively). After 60 days, there were significant differences in all chemical parameters examined (p<0.05). Corn stalk mixed with broccoli waste (CSBW) had the lowest DM loss (2.23%), and the CSOW group had the lowest NDF and ADF content and highest *in vitro* DM digestibility. CSBW had the lowest pH and ammonia nitrogen content, but the highest lactic acid/acetic acid ratio among the treatment groups. CSOW had the highest lactic acid content (2.27% of DM). The microbial contents of each group differed only in lactic acid bacteria counts before and after ensiling, showing a slight increase (p>0.05) and significant decreases in yeast and mold counts (p<0.05) after ensiling.

**Conclusion:**

These findings confirmed that mixing various FVW materials, particularly orange waste, with corn stalks improved the nutritional value of silage. Adding broccoli waste resulted in better fermentation quality than the addition of other FVW materials.

## INTRODUCTION

Corn (*Zea mays* L.) is one of the most important and widely cultivated cereal crops in the world, and it plays a critical role in agriculture [1]. Farmers usually leave corn stalks in the field for some time after harvesting the ears, as this allows the stalks to gradually decompose and provide nutrients to the soil, which can benefit crop growth in the next season [2]. Due to the scarcity of animal feed [3], corn stalks can be used as a roughage source in livestock feed. To preserve nutritional content and improve storability, corn stalks are commonly made into silage [4], particularly in areas with heavy precipitation.

However, there are challenges associated with using corn stalks as silage. For example, due to the time spent in the field, the stalks are relatively dry and may require additional moisture for optimal fermentation during silage. In addition, due to the low sugar content of corn stalks, they may require longer fermentation times or additives to facilitate fermentation. These issues may be overcome by mixing the stalks with other moist, sugar-rich feed materials such as fruit and vegetable waste (FVW) [5].

FVW consists of wounded and diseased tissue, residual leaves, and secondary fruits removed during the production, harvesting, processing, transportation, and sale of vegetables and fruits to improve their commercial appeal; FVW accounts for more than 30% of vegetable and fruit output [6]. As of 2021, the vegetable crop acreage in China was 21.99 million ha, the total vegetable production volume was 77.55 million tons, the fruit production acreage was approximately 13 million ha, and the total fruit production volume was nearly 300 million metric tons, with FVW exceeding 300 million tons [7–10]. FVW is rich in nutrients and has high moisture content, which can supplement the moisture content of delayed-harvest corn stalks, making them more suitable for silage.

Previous studies on FVW silage have mainly examined the impact of feeding FVW to ruminants as roughage or changes in its nutrients after ensiling [11]. By contrast, few studies have investigated mixing FVW with corn stalks for silage or the types of FVW that are most suitable for preparing mixed silage. Therefore, we mixed the four most common types of FVW (apple, orange, broccoli, and Chinese cabbage waste) with corn stalks and evaluated the quality of the resulting silage according to sensory evaluation and laboratory analyses of the chemical parameters, microbial contents, and fermentation of FVW.

## MATERIALS AND METHODS

### Material preparation and ensiling

Corn (*Zea mays* L.) was sown on May 5, 2022, at Seoul National University, Pyeongchang Campus, Gangwon-do, South Korea (37°32′46.10″N, 128°26′17.90″E). The ears were harvested on August 18, 2022, and the stalks were left in the field for 20 days and then mixed with four types of FVW (apple, orange, broccoli, and Chinese cabbage waste) at 3% of fresh matter (FM) for silage. No FVW was added to the control. Each FVW material was prepared 1 h before the stalks were harvested. Fruit waste residue was obtained after juicing fruit with a juicer (HN-SBF11; Hurom, Inc., Gangnam, Korea). Vegetable waste was collected from farms and cut into small pieces (2 to 3 cm). Corn stalks were chopped into pieces after harvesting (2 to 3 cm) and mixed with the FVW.

The mixed stalks and FVW were placed into 20-L silo buckets, compacted, and sealed. Each silo bucket held approximately 10 kg of raw material. Treatments were performed with three replicates. The buckets were stored in a cool, ventilated warehouse at approximately 25°C and opened after 60 days. Samples were collected to analyze the chemical composition, bacterial community population, and fermentation characteristics of the FVW.

### Chemical and microbiological analyses

The dry matter (DM) content of fresh and silage samples was determined after oven-drying at 65°C for 3 days (72 h). The dried samples were ground into powder using a grinder (Thomas Scientific, Swedesboro, NJ, USA) and passed through a 0.20-mm sieve for chemical analysis. The water-soluble carbohydrate (WSC) content was determined using a modification of the anthrone method proposed by Yemm and Willis [12]. Crude protein (CP) content was analyzed according to the method of the Association of Official Analytical Chemists (AOAC) [13]. Acid detergent fiber (ADF) and neutral detergent fiber (NDF) content was determined using an Ankom2000 fiber analyzer (Ankom Technology, Macedon, NY, USA) according to the method of Van Soest et al [14]. *In vitro* DM digestibility (IVDMD) was determined using the two-stage technique [15]. Samples (0.5±0.09 g) were incubated in buffer solutions (1,330 mL of buffer A and 266 mL of buffer B) in nylon filter bags (50 mm×55 mm; Ankom F57; Ankom Technology, USA) at 39°C overnight. Then, 400 mL of ruminal gastric juice from cattle was added and incubated at 39°C for a further 48 h. Analyses were performed as described for NDF.

The total digestible nutrient (TDN) content and relative feed value (RFV) are important indicators used to describe the nutritive value of feedstuffs, and were calculated as follows [16,17]:


$$\begin{array}{l} \text{TDN: TDN}\%=87.84-0.70\times \text{ADF}\%\\ \text{RFV: DDM}\%=88.9-0.779\times \text{ADF}\%\\ \text{DMI}\%=120/\text{NDF}\%\\ \text{RFV}=(\text{DMI}\%\times \;\text{DDM}\%)/1.29 \end{array}$$

Samples (10 g) of fresh material and silage were homogenized in 90 mL of sterile saline (0.85% NaCl solution) and shaken for 1 h. The extract was serially diluted from 1:10^2^ to 1:10^5^ with 0.85% NaCl solution, and 50-μL aliquots of each FVW type were spread evenly on the surfaces of agar plates and placed in an incubator. Lactic acid bacteria (LAB) populations were counted during incubation in De Man, Rogosa, and Sharpe agar (MRS) medium at 37°C for 24 to 48 h. The total microorganism (TM) population was counted using plate count agar medium under incubation at 37°C for 48 to 72 h. Yeast and mold (YM) counts were determined using potato dextrose agar medium under incubation at 25°C for 48 to 72 h. After culture, colony-forming units per gram (cfu/g) of microorganisms on the agar plates were counted and log_10_-transformed according to the dilution factor.

### Fermentation analysis

Silage samples (10 g) were blended in 90 mL of distilled water in Erlenmeyer flasks, which were sealed and shaken for 1 h. Samples were stored at 4°C for 24 h, and then filtered using filter paper (Whatman no. 6; Advantec MFS, Dublin, CA, USA). The silage filtrates were used for pH analysis using a pH meter (AB 150; Fisher Scientific Co., Pittsburgh, PA, USA), ammonia nitrogen (NH_3_-N) content determination by a modification of the phenol–hypochlorite reaction method [18], and lactic acid (LA), acetic acid (AA), propionic acid, and butyric acid content determination by high-performance liquid chromatography (HPLC) using an Agilent HPLC 1260 system with an Agilent Hi-Plex H column (7.7 mm×300 mm, 8 μm, p/n PL1170-6830; Agilent Technologies, Santa Clara, CA, USA), with a mobile phase consisting of 0.005 M H_2_SO_4_ at a flow rate of 0.7 mL/min.

### Statistical analyses

The data were completely randomized with 5×3 factorial permutation and analyzed using a general linear model in SPSS v26 (SPSS Inc., Chicago, IL, USA). The effects of FVW were analyzed by one-way analysis of variance. All graphics were prepared using GraphPad Prism v8.0.2 (GraphPad Software, San Diego, CA, USA). Differences among treatments were examined by Fisher’s least significant difference test. In all analyses, p<0.05 was taken to indicate statistical significance.

## RESULTS

### Microbial counts and chemical composition of raw materials

There were significant differences in all chemical composition parameters (DM, WSC, CP, NDF, ADF, and IVDMD) among apple, orange, broccoli, and Chinese cabbage waste (all p< 0.05) (Table 1). Apple waste had the highest DM content (>25.0%), followed in decreasing order by orange, Chinese cabbage, and broccoli waste. Apple waste had the highest WSC content (>50.0% of DM) followed by orange waste (39.29% of DM). Although broccoli waste had the lowest WSC content (12.18% of DM), it had the highest CP content (25.38% of DM), followed by Chinese cabbage waste (>20.0% of DM). Apple waste had the lowest CP content (3.19% of DM). Chinese cabbage waste had the highest NDF content (29.28% of DM), with no significant differences among the other groups. Orange waste had the lowest ADF content (<15.0% of DM), with no significant differences among the other groups. Broccoli waste had the highest IVDMD content (96.06% of DM), which was not significantly different from orange and Chinese cabbage waste (94.55% and 94.56% of DM, respectively), but significantly higher than that of apple waste (87.98% of DM, p<0.05).

The microorganism counts and chemical composition of corn stalks mixed with different FVW before ensiling are presented in Table 2 and 3. There were no significant differences among groups in TM or YM counts, which had averages of approximately 6.7 and 5.5 log_10_ cfu/g FM, respectively. However, there were significant differences in LAB populations among treatment groups (p<0.05), which ranged from 4.93 to 5.57 log_10_ cfu/g FM and varied in the order apple> control>orange>broccoli>Chinese cabbage.

The different treatments did not affect CP or IVDMD content, which had average values of approximately 4.4% and 68.0% of DM, respectively. All treatment groups showed significant differences in DM, WSC, NDF, and ADF content as well as the two feed nutritional value indicators, TDN and RFV (all p<0.05). Compared with the control group, FVW treatment groups had lower NDF and ADF content and higher TDN and RFV values. The corn stalk mixed with orange waste (CSOW) treatment group had the highest DM, TDN, and RFV content and the lowest NDF and ADF content, whereas the corn stalk mixed with broccoli waste (CSBW) treatment group had the lowest WSC content.

### Chemical compositions and fermentation states of corn stalk silage

The chemical compositions of corn stalk silage mixed with different FVW types after 60 days of ensiling are shown in Table 4. RFV and the other chemical parameters showed significant differences among treatment groups (all p<0.05). In comparison with the raw materials, DM content decreased in all FVW treatment groups, with the greatest reduction of approximately 13.0% in the CSOW treatment group. After ensiling, there were significant differences in DM loss associated with each treatment (all p<0.05). DM loss was greatest in the CSOW treatment group (>12.0%) and lowest in the CSBW treatment group (~2.0%). WSC content showed a sharp decline in all groups, which was most pronounced in the CSAW and CSBW treatment groups. By contrast, CP content showed little change. IVDMD decreased in all treatment groups, and all groups had higher NDF and ADF content than the control group. However, the CSOW treatment group also showed the lowest ADF and NDF content and greater DM digestibility. TDN and RFV content was also higher in the CSOW treatment group than in the other treatment groups, although TDN and RFV content decreased in all groups after ensiling.

The fermentation indicators pH, NH_3_-N, and organic acid content are shown in Table 5. The pH of raw materials showed no significant differences among treatments. All indicators showed significant changes in all treatment groups after ensiling (p<0.05). The pH decreased from 5.1 to 3.7, with the CSBW treatment group showing the lowest pH. In comparison with the control group, the CSBW treatment group had lower NH_3_-N content, which was not significantly different from that of the other treatment groups. LA content was significantly higher in the CSOW treatment group than in the CSCW treatment group (p<0.05). Except for the CSOW treatment, all treatment groups showed similar AA content to the control group. The LA/AA ratio was also significantly different among the treatment groups, with the control group and CSBW treatment group showing the highest ratios (>3.0).

### Microbial population of corn stalk silage

Table 6 shows the microbial compositions of corn stalk silage with different FVW types after ensiling. Similar to the raw materials, LAB counts were significantly different among all treatment groups (p<0.05). The TM count was similar before and after ensiling, and the YM count decreased after 60 days. LAB counts were not markedly different from their respective raw materials, and the CSAW treatment group had a significantly higher LAB count than all other treatments (p<0.05).

## DISCUSSION

### Chemical composition of silage consisting of corn stalks and different fruit and vegetable waste types

Larger TDN and RFV values in animal feedstuffs indicate better feed quality [19]. Before ensiling, each treatment group had higher nutritional value than the control, with the highest feed value in the CSOW treatment group, perhaps because orange waste has lower fiber content than the other FVW types examined in this study. DM content, which is the key to successful silage fermentation, was higher in the CSOW treatment group than in the other groups. In general, good silage fermentation requires WSC content of at least 6.0% to 8.0% of DM [20]. In this study, the WSC content of each group was greater than 14.0% of DM. As apple, orange, and Chinese cabbage waste showed high WSC content, the CSAW, CSOW, and CSCW treatment groups also had higher WSC content (>25.0%), indicating good fermentation conditions.

Compared with the raw materials, the TDN, RFV, and IVDMD content decreased after 60 days of storage. This result may have been due to heterolactic fermentation caused by high FVW moisture content [21]. The fermentation of heterofermentative LAB or yeast can produce mannitol and ethanol, during which a portion of soluble nutrients can form gas or be lost due to osmotic loss, resulting in a relative increase in fiber content [22]. Good silage requires lower fiber content and higher feed value. DM loss is among the parameters most clearly associated with changes in nutrients before and after ensiling, with greater DM loss indicating greater nutrient loss [23]. DM loss was significantly greater in the CSOW treatment group (>12.0%) than in the other groups, whereas the CSBW treatment group showed the lowest DM loss (<3.0%). This result may have been due to heterolactic fermentation in the CSOW treatment group, as DM loss is related to heterofermentative LAB and heterofermentation is accompanied by high DM loss [24]. This hypothesis was confirmed by the high AA content and low LA/AA value (<3.0) in the CSOW treatment group. WSC can be utilized by microorganisms as a substrate for fermentation to promote their growth [25], and can promote LAB growth; greater WSC consumption has been shown to be related to greater LAB activity [26]. The CSAW treatment group had the highest LAB count before ensiling and showed the greatest reduction in WSC content after storage, with the highest LAB activity among all treatment groups. The CP content increased in the control, CSAW, CSBW, and CSCW treatment groups after ensiling, indicating that these treatments retained nutrients well during the ensiling process [27].

Each treatment group showed more favorable chemical parameters compared with the control group. The CSOW treatment showed the best feed value but the greatest loss of nutritional value after ensiling. Therefore, adding orange waste to corn stalks can yield high-quality feed, which is consistent with a previous study that reported that oranges and other citrus fruits are rich in pectin and highly degradable NDF [28]. When mixed with straw as a basic raw material, orange waste can improve the nutritional structure and increase the digestibility of crops; however, its nutrients are easily lost during the ensiling process.

### Fermentation state and microorganism population of mixed corn stalk silage

High homofermentative LAB content allows homolactic fermentation to dominate in the early stages of storage, producing good fermentation [29]. LA and AA content levels are important indicators of silage fermentation quality, and the dominance of homolactic fermentation increases LA content and causes rapid pH decline [30]. The LA/AA ratio can be used as an index of the relative importance of homolactic fermentation, with LA/AA >3 indicating homolactic fermentation dominance [31]. In this study, there were no significant differences in total microorganisms or YM populations among treatment groups before ensiling, whereas LAB populations were higher in the control and CSAW treatment groups than in all other treatments. The CSCW treatment group had the lowest LAB count, the highest pH after ensiling, the lowest LA content, and the lowest LA/AA ratio (<3.0). These results indicated that the CSCW treatment group had poorer fermentation quality than all other groups. Moreover, all groups except the control and CSBW treatment groups showed LA/AA <3, indicating heterolactic fermentation.

However, the CSAW treatment group had a higher LAB count than the control group before ensiling, but did not show good fermentation properties, perhaps due to a higher proportion of heterofermentative LAB strains and a lower proportion of homofermentative LAB strains, which promote homolactic fermentation in the LAB population. This condition would have led to less LA accumulation. Similarly, homofermentative LAB has been shown to ensure rapid and vigorous fermentation by promoting LA production [32]. The CSBW treatment group had better fermentation properties than the other treatments. Although the LAB count was not high for raw CSBW materials, the CSBW treatment group had higher DM content and lower DM loss; therefore, we estimated that this treatment group had a high proportion of homofermentative LAB strains, which inhibit clostridial fermentation and reduce silage nutrient loss [33], leading to good preservation.

Although heterofermentative LAB does not accumulate LA as strongly as homofermentative LAB [34], it also produces volatile short-chain fatty acids that effectively inhibit the growth of aerobic microorganisms such as yeasts and molds, and improve aerobic stability. In this study, the YM counts of each group decreased significantly after ensiling, whereas LAB and TM counts were not similarly affected. Thus, all treatments in this study exhibited some heterolactic fermentation, implying high proportions of heterofermentative LAB strains in each treatment group, which led to a lack of significant LA accumulation, but effective inhibition of YM growth.

NH3-N is an index that reflects the degree of CP degradation during the ensiling process, and is negatively correlated with silage quality [35]. An Agricultural and Food Research Council [36] report indicated that good-quality silage has NH_3_-N content below 10% of total N. In the present study, this requirement was fulfilled in all groups, indicating that all treatments led to good silage quality. NH_3_-N content was lowest in the CSBW treatment group, indicating better silage quality, perhaps due to the LAB strains present. Homofermentative LAB can inhibit the hydrolysis of CP and reduce NH_3_-N content, whereas NH_3_-N content is not affected by heterofermentative LAB [37]. Our results also suggested that microorganisms in the CSBW treatment group led to good fermentation quality. Further studies are required to analyze the specific microbial types present under CSBW treatment.

## CONCLUSION

In this study, we found that mixing various types of FVW, particularly orange waste, with corn stalks can improve the value of silage. The fermentation quality of each FVW treatment group was affected by moisture content and the types of LAB present. Fermentation quality was improved by the addition of broccoli waste, which had the lowest DM content among the FVW types examined. Therefore, we recommend adding FVW to corn stalk silage, specifically broccoli waste for producing silage for long-term storage.

## Figures and Tables

**Table 1:** Chemical compositions of different fruit and vegetable wastes before ensiling

Waste	DM	WSC	CP	NDF	ADF	IVDMD
	
	%	% DM				
Apple	25.04^a^	50.76^a^	3.19^d^	26.88^ab^	16.24^ab^	87.98^b^
Orange	22.65^b^	39.29^b^	5.89^c^	25.52^b^	14.95^b^	94.55^a^
Broccoli	5.30^d^	12.18^d^	25.38^a^	26.28^b^	18.07^a^	96.06^a^
Chinese cabbage	6.56^c^	20.48^c^	20.41^b^	29.28^a^	18.05^a^	94.56^a^
SEM	5.20	8.77	5.42	0.81	0.76	1.80
p-value	<0.05	<0.05	<0.05	<0.05	<0.05	<0.05

DM, dry matter; WSC, water-soluble carbohydrates; CP, crude protein; NDF, neutral detergent fiber; ADF, acid detergent fiber; IVDMD, in vitro dry matter digestibility; SEM, standard of error mean.

a–d Values with different letters in the same column are significantly different (p<0.05).

**Table 2:** Microorganism population of corn stalks with different fruit and vegetable wastes before ensiling

Treatment	LAB	TM	YM

	Log_10_ cfu/g FM		
Control	5.41^ab^	6.81	5.69
CSAW	5.57^a^	6.70	5.59
CSOW	5.31^b^	6.64	5.47
CSBW	5.15^bc^	6.66	5.24
CSCW	4.93^c^	6.82	5.67
SEM	0.11	0.04	0.08
p-value	<0.05	0.11	0.28

LAB, lactic acid bacteria; TM, total microorganisms; YM, yeasts and molds; FM, fresh matter; CSAW, corn stalks with 3% apple waste; CSOW, corn stalks with 3% orange waste; CSBW, corn stalks with 3% broccoli waste; CSCW, corn stalks with 3% Chinese cabbage waste; SEM, standard of error mean.

a–c Means with different lower-case letters differ (p<0.05).

**Table 3:** Chemical compositions of corn stalks with different fruit and vegetable wastes before ensiling

Treatment	DM	WSC	CP	NDF	ADF	IVDMD	TDN	RFV
	
	%	% DM						
Control	28.54^a^	14.15^b^	4.30	52.11^a^	29.02^a^	66.27	67.53^c^	118^c^
CSAW	28.11^ab^	31.19^a^	4.29	49.29^bc^	27.42^bc^	69.52	68.65^ab^	128^b^
CSOW	28.77^a^	29.42^a^	4.34	47.78^c^	26.62^c^	70.10	69.21^a^	133^a^
CSBW	26.19^c^	15.87^b^	4.54	49.68^b^	27.82^b^	71.52	68.37^b^	126^b^
CSCW	27.21^b^	27.56^a^	4.48	49.85^b^	27.61^bc^	70.35	68.51^ab^	126^b^
SEM	0.48	3.58	0.04	0.70	0.39	0.88	0.41	2.30
p-value	<0.05	<0.05	0.60	<0.05	<0.05	0.25	<0.05	<0.05

DM, dry matter; WSC, water-soluble carbohydrates; CP, crude protein; NDF, neutral detergent fiber; ADF, acid detergent fiber; IVDMD, in vitro dry matter digestibility; TDN, total digestible nutrients; RFV, relative feed value; CSAW, corn stalks with 3% apple waste; CSOW, corn stalks with 3% orange waste; CSBW, corn stalks with 3% broccoli waste; CSCW, corn stalks with 3% Chinese cabbage waste; SEM, standard of error mean.

a–c Means with different lower-case letters differ (p<0.05).

**Table 4:** Chemical compositions of corn stalks silage with different fruit and vegetable wastes after 60 days

Treatment	DM	DM loss	WSC	CP	NDF	ADF	IVDMD	TDN	RFV
	
	%		% DM						
Control	25.80^a^	9.57^c^	3.91^a^	4.39^b^	51.79^a^	29.51^a^	59.79^b^	67.18	119^c^
CSAW	25.30^b^	9.99^b^	3.09^b^	4.41^b^	51.57^a^	29.44^a^	65.01^a^	67.23	119^bc^
CSOW	25.17^b^	12.52^a^	3.92^a^	4.47^ab^	50.49^b^	28.77^b^	65.98^a^	67.70	123^a^
CSBW	25.60^a^	2.23^e^	3.40^b^	4.69^a^	51.50^ab^	29.21^ab^	65.99^a^	67.40	120^abc^
CSCW	24.69^b^	9.24^d^	3.31^b^	4.63^a^	50.46^b^	29.25^ab^	62.38^b^	67.36	122^ab^
SEM	0.19	0.02	0.17	0.06	0.28	0.13	1.21	0.45	0.82
p-value	<0.05	<0.05	<0.05	<0.05	<0.05	<0.05	<0.05	0.14	<0.05

DM, dry matter; WSC, water-soluble carbohydrates; CP, crude protein; NDF, neutral detergent fiber; ADF, acid detergent fiber; IVDMD, in vitro dry matter digestibility; TDN, total digestible nutrients; RFV, relative feed value; CSAW, corn stalks with 3% apple waste; CSOW, corn stalks with 3% orange waste; CSBW, corn stalks with 3% broccoli waste; CSCW, corn stalks with 3% Chinese cabbage waste; SEM, standard of error mean.

a–e Means with different lower-case letters differ (p<0.05).

**Table 5:** pH, NH_3_-N, and organic acids of corn stalks silage with different types of fruit and vegetable wastes after 60 days

Treatment	pH		NH_3_-N % total N	LA	AA	PA	BA	LA/AA
	
	Raw material	Silage		% DM				
Control	5.10	3.65^bc^	7.41^a^	2.01^b^	0.66^b^	ND	ND	3.05^a^
CSAW	5.10	3.66^b^	7.67^a^	1.96^bc^	0.70^b^	ND	ND	2.82^b^
CSOW	5.12	3.65^bc^	7.57^a^	2.27^a^	0.82^a^	ND	ND	2.77^bc^
CSBW	5.12	3.64^c^	6.23^b^	2.00^b^	0.65^b^	ND	ND	3.07^a^
CSCW	5.06	3.70^a^	7.58^a^	1.87^c^	0.69^b^	ND	ND	2.71^c^
SEM	0.01	0.01	0.27	0.07	0.03	ND	ND	0.07
p-value	0.83	<0.05	<0.05	<0.05	<0.05	ND	ND	<0.05

NH_3_-N, ammonia nitrogen; LA, lactic acid; AA, acetic acid; PA, propionic acid; BA, butyric acid; DM, dry matter; ND, not detected; CSAW, corn stalks with 3% apple waste; CSOW, corn stalks with 3% orange waste; CSBW, corn stalks with 3% broccoli waste; CSCW, corn stalks with 3% Chinese cabbage waste; SEM, standard of error mean.

a–c Means with different lower-case letters differ (p<0.05).

**Table 6:** Microorganism population of corn stalks with different fruit and vegetable wastes after 60 days

Treatment	LAB	TM	YM

	Log_10_ cfu/g FM		
Control	5.39^b^	6.29	4.22
CSAW	5.56^a^	6.38	4.08
CSOW	5.43^b^	6.31	3.91
CSBW	5.31^c^	6.30	4.01
CSCW	5.19^d^	6.27	4.31
SEM	0.04	0.13	0.35
p-value	<0.05	0.39	0.12

LAB, lactic acid bacteria; TM, total microorganisms; YM, yeasts and molds; FM, fresh matter; CSAW, corn stalks with 3% apple waste; CSOW, corn stalks with 3% orange waste; CSBW, corn stalks with 3% broccoli waste; CSCW, corn stalks with 3% Chinese cabbage waste; SEM, standard of error mean.

a–d Means with different lower-case letters differ (p<0.05).
